# Supplementary material for: An optimised GAS-pharyngeal cell biofilm model
Source: Sci Rep. 2021 Apr 15;11:8200. doi: 10.1038/s41598-021-87377-7 (PMC8050266; doi:10.1038/s41598-021-87377-7)
Supplement: Supplementary file 1 — Supplementary Information. [file 41598_2021_87377_MOESM1_ESM.docx]

**Supplementary Materials:**

**Supplementary Material 1:**

**Table 1. Crystal violet staining data (OD_540nm_) obtained for Detroit 562 pharyngeal cell monolayers (without GAS biofilm) that were used as background blanks and subtracted from wells containing GAS biofilm formed atop monolayers for Fig. 3.** Data represents mean ± SEM; n = 3 biological replicates, with 3 technical replicates each.

| **Replicate** | **Crystal violet stained**  **Detroit 562 pharyngeal cell monolayers (OD_540nm_)** |
| --- | --- |
| **1** | 0.15 ± 0.03 |
| **2** | 0.15 ± 0.06 |
| **3** | 0.20 ± 0.03 |
